# Evaluating Established Methods for Rumen 16S rRNA Amplicon Sequencing With Mock Microbial Populations

**DOI:** 10.3389/fmicb.2018.01365

**Published:** 2018-06-25

**Authors:** Emily McGovern, Sinéad M. Waters, Gordon Blackshields, Matthew S. McCabe

**Affiliations:** ^1^Animal and Bioscience Research Department, Animal and Grassland Research and Innovation Centre, Teagasc, Carlow, Ireland; ^2^UCD College of Health and Agricultural Sciences, University College Dublin, Dublin, Ireland

**Keywords:** mock community, rumen, amplicon, PCR, microbiota, phylogenetic analysis

## Abstract

The rumen microbiome scientific community has utilized amplicon sequencing as an aid in identifying potential community compositional trends that could be used as an estimation of various production and performance traits including methane emission, animal protein production efficiency, and ruminant health status. In order to translate rumen microbiome studies into executable application, there is a need for experimental and analytical concordance within the community. The objective of this study was to assess these factors in relation to selected currently established methods for 16S phylogenetic community analysis on a microbial community standard (MC) and a DNA standard (DS; ZymoBIOMICS^TM^). DNA was extracted from MC using the RBBC method commonly used for microbial DNA extraction from rumen digesta samples. 16S rRNA amplicon libraries were generated for the MC and DS using primers routinely used for rumen bacterial and archaeal community analysis. The primers targeted the V4 and V3–V4 region of the 16S rRNA gene and samples were subjected to both 20 and 28 polymerase chain reaction (PCR) cycles under identical cycle conditions. Sequencing was conducted using the Illumina MiSeq platform. As the bacteria contained in the microbial mock community were well-classified species, and for ease of explanation, we used the results of the Basic Local Alignment Search Tool classification to assess the DNA, PCR cycle number, and primer type. Sequence classification methodology was assessed independently. Spearman’s correlation analysis indicated that utilizing the repeated bead beating and column method for DNA extraction in combination with primers targeting the 16S rRNA gene using 20 first-round PCR cycles was sufficient for amplicon sequencing to generate a relatively accurate depiction of the bacterial communities present in rumen samples. These results also emphasize the requirement to develop and utilize positive mock community controls for all rumen microbiomic studies in order to discern errors which may arise at any step during a next-generation sequencing protocol.

## Introduction

The rumen microbiota enables the ruminant to digest cellulose-rich feedstuff and to convert them into a wide range of metabolites that are used by both microbes for their own proliferation and also for the supply of nutrients to the host animal. This symbiotic relationship between the rumen microbiota and host is vital to the ruminant’s survival and performance (Saleem et al., 2012). The diverse microbial community adapts to a wide array of dietary feedstuffs and management strategies (Knapp et al., 2014). Elucidating the rumen microbiome composition, adaptation, and function is of international interest due to its implications, ranging from climate change to applied animal production and animal health (McCann et al., 2014).

Classical knowledge of rumen microbiology was previously based on anaerobic, culture-dependent methods. However, in recent years, the development of next-generation sequencing (NGS) has permitted in-depth sequencing and data analysis of various types of environmental samples (Stulberg et al., 2016). Amplicon sequencing targeting the 16S rRNA in bacteria and archaea remains the most cost-effective and facile tool to provide valuable phylogenetic information for the comparison of microbial diversity in large numbers of environmental samples. Numerous research groups investigating the rumen microbiome have utilized amplicon sequencing as an aid in identifying potential community compositional trends that could be used as an estimation of various performance traits, including methane emission, animal protein production, and ruminant health status (Hook et al., 2011; Petri et al., 2013; Shi et al., 2014; Roehe et al., 2016; Shabat et al., 2016). It has been identified that there is no standardization between amplicon studies (Sinha et al., 2017) and it has been emphasized that this lack of standardization is causing issues with research replication as well as problems translating research into applications (Sinha et al., 2015). The assessment of US microbiome research also identified a need for standardized protocols between the microbiome research communities in order to facilitate comparisons between studies (Stulberg et al., 2016).

The method utilized for DNA extraction can lead to dramatic differences in microbial output composition (Henderson et al., 2013; Brooks et al., 2015). Hence, validating extraction methods with a mock microbial community is an absolute necessity to ensure accurate representation of microbial communities in samples. The extraction method assessed in the current study is the repeated bead beating and column (RBBC) method (Yu and Morrison, 2004) as it is a common method utilized for extraction of DNA from rumen contents (Henderson et al., 2013; Wallace et al., 2014; McCabe et al., 2015). However, this DNA extraction method has not been validated with a mock microbial community standard (MC) to verify its efficiency at extracting accurate representative quantities of DNA from both Gram-positive and Gram-negative bacteria.

Comparisons of the rumen bacterial and archaeal community compositions are typically investigated by sequencing one or more of the nine different hypervariable regions of the 16S rRNA gene, due to conservation across the domains of bacteria and archaea (Pace, 1997; Tringe and Hugenholtz, 2008). Most NGS amplicon studies in recent years have been conducted on the Illumina MiSeq as this platform gives the highest number of reads at the lowest cost. However, the length of reliable high-quality reads remains at 250 bp for the forward read and approximately 230 bp for the reverse read. Consequently, this allows sequencing of only one or two 16S rRNA variable regions which limits the analysis to genus level identification at best. Despite this limited resolution, amplicon sequencing on the Illumina MiSeq is a very useful approach for screening large numbers of complex microbial community samples to identify samples that show major perturbations in the composition of microbial genera. The choice of a specific hyper variable region can affect both the accuracy and specificity of phylogenetic assignment and the community diversity estimation (Liu et al., 2008; Youssef et al., 2009). In the current study, we tested two commonly used primer pairs – 515F/806R (Caporaso et al., 2011) and Pro341F/Pro805R (Takahashi et al., 2014) – that both simultaneously amplify bacteria and archaea. It has been previously shown that simultaneous amplification of archaea and bacteria using multi-domain specific universal primers may not be as accurate as separate analysis of the archaeal and bacterial domain (Klindworth et al., 2013; Fischer et al., 2016). However, simultaneous amplification of archaea and bacteria is particularly appropriate for rumen microbiomic diversity studies as there are major efforts to develop strategies in cattle and sheep production that reduce the numbers of rumen archaea relative to bacteria (Roehe et al., 2016). It also facilitates investigation into relationships between microbial taxa from both archaeal and bacterial domains in a convenient, one step-approach, for example, McCabe et al. (2015) previously deciphered an inverse relationship between a member of *Methanobrevibacter gottschalkii* clade and uncharacterized Succinivibrionaceae species utilizing this method. The primer pair Pro341F/Pro805R targets V3–V4 and is a subsequent modification of the primers reported by Klindworth et al. (2013). Pro341F/Pro805R was reported to generate microbial profiles that were more accurate than those obtained with other commonly used bacterial and archaeal primer pairs (Takahashi et al., 2014). The primer pair 515F/806R targets the V4 region of the 16S rRNA gene and was previously validated with a mock community. However, Caporaso et al. (2011) utilized an alternative amplification protocol with a single round of polymerase chain reaction (PCR) rather than the two-step PCR protocol used in this investigation.

Polymerase chain reaction cycle conditions are another contentious issue that can cause misrepresentation of microbial community composition. A two-step PCR protocol is routinely used to initially amplify the hypervariable region of interest and, second, to barcode samples in order to facilitate multiplex sequencing (Illumina, 2013). It was our aim to assess our current methodology (McCabe et al., 2015) against a lower number of PCR cycle conditions for the first amplicon generation step. Superfluous PCR cycles can lead to the formation of PCR by-products (Qiu et al., 2001) such as chimeras (Wang and Wang, 1997), heteroduplexes, and single-stranded DNA molecules (Jensen et al., 1993). The classification of sequences is yet another element of consideration for 16S rRNA phylogenetic analysis of bacterial and archaeal communities. For the present study, we used multiple sequence alignment with Basic Local Alignment Search Tool (BLAST) to identify species from DNA that were extracted from mock community cells. This BLAST approach was successful as the mock community is composed of only eight well-characterized bacterial species and two well-characterized yeast species. We also tested the Ribosomal Database Project (RDP) classifier (Wang et al., 2007), which is a popular naïve Bayesian classifier (Puniya et al., 2015) for taxonomic assignment of rumen bacterial and archaeal species (McCabe et al., 2015; Meale et al., 2016; Popova et al., 2017). The taxonomic classifier can be used independently and can be encapsulated into other programs and pipelines. The overall objective of the study was to evaluate standard methodology frequently utilized for phylogenetic surveys of the rumen bacterial and archaeal community and assess whether it is giving an accurate representation of microbial communities using a commercially available mock MC and a DNA standard (DS). The mock microbial community utilized for this evaluation is commercially available and is composed of five Gram-positive bacteria (i.e., difficult to lyze): *Lactobacillus fermentum*, *Enterococcus faecalis*, *Staphylococcus aureus*, *Listeria monocytogenes*, and *Bacillus subtilis*, three Gram-negative bacteria: *Escherichia coli*, *Salmonella enterica*, and *Pseudomonas aeruginosa*, and two yeast species: *Saccharomyces cerevisiae* and *Cryptococcus neoformans*.

## Materials and Methods

### DNA Extraction

In order to replicate the DNA extraction protocol identically to our rumen extraction protocol, the ZymoBIOMICS^TM^ Microbial Community Standard (Zymo Research Corp., Irvine, CA, United States) was diluted in a 2-mL microfuge tube (DNA/RNA/DNase/RNase-free; Merck KGaA, Darmstadt, Germany) with molecular grade phosphate buffered saline (PBS; Merck KGaA, Darmstadt, Germany), and the tube and its contents were snap frozen in liquid nitrogen. The frozen tube contents were then homogenized to a fine frozen powder under liquid nitrogen using a pestle and mortar and the frozen powder was stored in a 2-mL microfuge tube at -80°C. Approximately 250 mg homogenized frozen powder was used for DNA extraction from the mock community. DNA was also extracted from 250 mg of homogenized frozen rumen solid digesta powder sample and molecular grade water as a positive and a negative control (NC), respectively. DNA was extracted using the RBBC method (Yu and Morrison, 2004). The DNA quality (i.e., approximation of high molecular weight DNA:degraded DNA and presence/absence of RNA) was assessed on an agarose gel at a normalized concentration of 100 ng/μL. DNA concentration and purity were assessed with two consecutive readings on the Nanodrop 1000 spectrophotometer.

### 16S rRNA Gene Amplicon Library Preparation

Five replicate amplicon libraries were generated for both the ZymoBIOMICS^TM^ Microbial Community Standard cells (MC; Zymo Research Corp., Irvine, CA, United States), which were extracted using the RBBC extraction method (Yu and Morrison (2004), and the ZymoBIOMICS^TM^ Microbial Community Standard DNA (DS; Zymo Research Corp., Irvine, CA, United States). The ZymoBIOMICS^TM^ Microbial Community Standard DNA (DS) was generated at Zymo by extracting DNA from pure colonies of each of eight bacteria species and two yeast strains and combining the resulting 10 DNA extractions in carefully defined amounts (12% for each of the bacterial DNA extracts and 2% for each of the two yeast DNA extracts). Libraries were also generated for positive (rumen digesta) and negative extraction (molecular grade water) controls, respectively.

Polymerase chain reactions were performed for amplicon libraries with 20 ng of rumen DNA 515F/806R (V4; Caporaso et al., 2011) and Pro341F/Pro805R (V3–V4) primers (Takahashi et al., 2014) and designed with Nextera overhang adapters, using 1X KAPA HiFi HotStart ReadyMix DNA polymerase (Roche Diagnostics, West Sussex, United Kingdom). Both 515F/806R and Pro341F/Pro805R matched to all bacterial DNA targets within MC and DS with no mismatch. Two sets of cycle conditions were applied for the initial PCR (PCR1) stage:

(a) 95°C for 3 min, 20 cycles; 95°C for 30 s, 55°C for 30 s, 72°C for 30 s, and then 72°C for 5 min
(b) 95°C for 3 min, 28 cycles; 95°C for 30 s, 55°C for 30 s, 72°C for 30 s, and then 72°C for 5 min.

Amplicons were purified using the Qiaquick PCR Purification Kit (Qiagen, Manchester, United Kingdom). A second PCR step was used to attach dual indices and Illumina sequencing adapters using Nextera XT index kit (Illumina, San Diego, CA, United States). Cycle conditions were 95°C for 3 min, 8 cycles; 95°C for 30 s, 55°C for 30 s, 72°C for 30 s, and then 72°C for 5 min. Amplicon generation was validated through visualization on a 2% (w/v) agarose gel (Yu and Morrison, 2004). Amplicons were pooled in equal concentrations and gel purified to remove adapter dimers using the Qiagen Gel Extraction Kit (Qiagen, Manchester, United Kingdom). An extra purification with the QIAquick purification kit (Qiagen, Manchester, United Kingdom) was used to remove residual agarose. The pooled purified libraries were measured for purity and quantity on the Nanodrop 1000 and further quantified using the KAPA SYBR FAST universal kit with Illumina Primer Premix (Roche Diagnositics, West Sussex, United Kingdom). The library pool was then diluted and denatured as recommended by the Illumina MiSeq library preparation guide. Sequencing was conducted using 500 cycle MiSeq reagent kits (Illumina, San Diego, CA, United States).

Zymo also provided the fastq files (ZE) containing 16S rRNA sequencing data from their extraction of the microbial mock community with the ZymoBIOMCS^TM^ DNA Mini Kit (Zymo Research Corp., Irvine, CA, United States). These data had been generated with primers targeting V3–4 region and the amplicons were sequenced on Illumina MiSeq (2 × 250 bp; Illumina, San Diego, CA, United States).

### Sequence Analysis

Read 1 and Read 2 of 16S rRNA gene from all amplicons were quality checked with FASTQC (Andrews, 2010)^1^ and overlapping reads (>Q30) were merged using BBMerge from the BBMap package^2^. The merged sequence outputs were processed and classified using four different methods:

(1) A high throughput NCBI BLAST (version 2.5) of individual sequences of merged reads was conducted against the NCBI 16S ribosomal RNA database.
(2) Merged reads from all samples were also subsequently combined into a single dataset for processing with QIIME (version 1.9). Chimeric sequences were removed via USEARCH using the ChimeraSlayer GOLD database (Edgar et al., 2011). A combination of *de novo* and reference-based operational taxonomic unit (OTU) identification was carried out using the open reference calling method implemented within QIIME. Sequences were clustered at 97% identity into individual OTUs using UCLUST (Edgar, 2010). A single representative sequence from each clustered OTU was aligned to the Greengenes (version gg_13_8), RDP (version 16), SILVA (Silva_128_release), and NCBI 16S ribosomal RNA database independently. Taxonomic classification for each OTU was determined using the RDP classifier (version 2.12).
(3) Identical sequences were identified from merged sequence reads and a unique representative sequence of the original sequences was used for sequence classification. Classification was conducted with the R package; microclass, using the taxMachine tool and the contax_trim database. Merged ZE sequence reads were also classified using the RDP classifier (version 2.9) in R, with the contax_trim database.

### Statistical Analysis

Sequence data from amplicon libraries (*n* = 48) were compared to the theoretical composition of the Zymo standards. 16S rRNA gene abundance was calculated from theoretical genomic DNA composition with the following formula: 16S copy number = total genomic DNA (g) × unit conversion constant (bp/g)/genome size (bp) × 16S copy number per genome (Zymo, 2016). Relative abundance was calculated for identified sequences in each sample at genus level. The experiment was conducted as a 2 × 2 factorial design. MC and DS replicates for each primer set and both cycle conditions examined were analyzed as an experimental unit for statistical analysis (*n* = 5 per unit). Group means were calculated for MC and DS replicates R Studio (v3.1). Spearman’s correlation analysis was conducted with hmisc package R Studio (v3.1; Harrell, 2014). A power analysis was conducted prior to experimentation to ensure that technical replication was sufficient to deduce a significant correlation between the technical treatments examined in the current experiment and the theoretical composition of the mock community supplied by Zymo. This utilized a significance level of 0.05, an expected correlation of 0.7, and a power of 0.2 (Champely, 2015). Similarity was declared as significant if *P* < 0.05 and as a trend if 0.05 > *P* ≤ 0.1.

### Summary of Methods

DNA was extracted from MC using the RBBC method commonly used for microbial DNA extraction from rumen digesta samples. 16S rRNA amplicon libraries were generated for the MC and DS using primers routinely used for rumen bacterial and archaeal community analysis. The primers targeted the V4 and V3–V4 region of the 16S rRNA gene and samples were subjected to both 20 and 28 PCR cycles under identical cycle conditions. Sequencing was conducted using the Illumina MiSeq platform. As the bacteria contained in the microbial mock community were well-classified species, and for ease of explanation, we used the results of the BLAST classification to assess the DNA, PCR cycle number, and primer type. Sequence classification methodology was assessed independently.

## Results

### DNA Extraction

**FIGURE 1:**
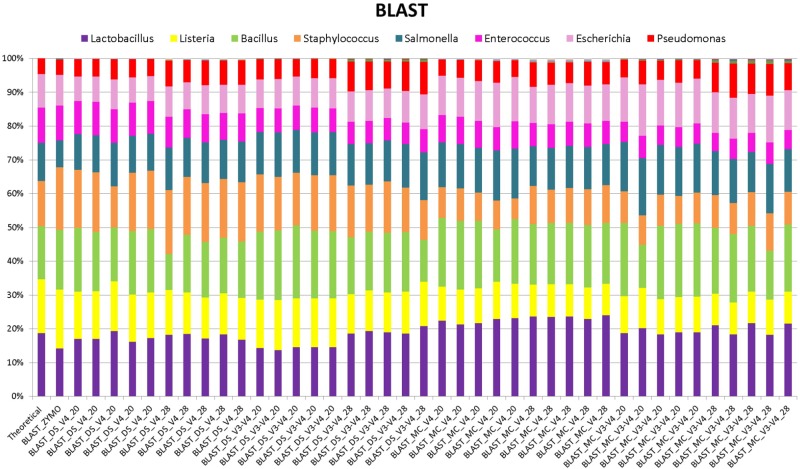
Stack plot comparing the relative abundance of the theoretical mock community compositions (Theoretical), Zymo 16S rRNA sequencing data (Zymo), mock community cells extracted using the RBBC method (MC), and mock community DNA (DS) amplified using primers targeting the V3–V4 and V4 region of the 16S rRNA gene with 20 and 28 cycles. Sequence taxonomy was classified through a high-throughput BLAST search against the NCBI 16S rRNA database.

**FIGURE 2:**
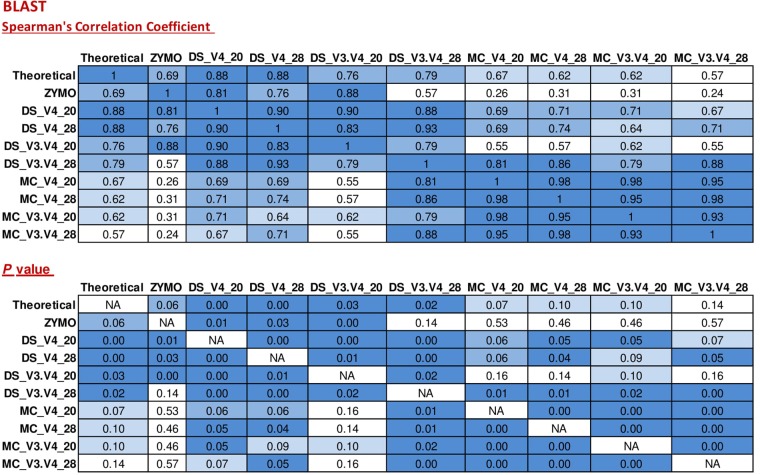
Correlation matrix of Spearman correlation coefficient values between the theoretical mock community compositions (Theoretical), Zymo 16S rRNA sequencing data (Zymo), mock community cells extracted using the RBBC method (MC), and mock community DNA (DS) amplified using primers targeting the V3–V4 and V4 region of the 16S rRNA gene with 20 and 28 cycles. Sequence taxonomy was classified through a high throughput BLAST search against the NCBI 16S rRNA database. Values with a positive significant correlation are illustrated with shaded color while insignificant correlations are blank.

When BLAST analysis was used to analyze the MiSeq amplicon sequence data, all eight expected bacterial genera were detected for all combinations of DNA extraction/primer pair/PCR cycle number (**Figure 1**). The largest effect on accuracy of genera relative abundances was DNA extraction method. As expected, DS (DNA that was pre-extracted by Zymo from each species then combined in equal amounts) showed significant (*P* < 0.05) Spearman correlation with the theoretical mock community relative abundances for all combinations of PCR cycle numbers and primers. MC (DNA extracted using RBBC method) showed non-significant Spearman correlations with theoretical mock community relative abundances (*P* > 0.05; **Figure 2**). The best result achieved for correlations between MC and the theoretical composition was a tendency toward significance (Spearman = 0.67, *P* = 0.07) when 20 PCR cycles were used in combination with the V4 primers.

Interestingly, correlation of ZE (FASTQ files from a MiSeq amplicon run conducted at Zymo using DNA they extracted from the mock community with their Zymo kit) with the theoretical mock community relative abundance values showed a Spearman correlation of 0.69 (*P* = 0.058) which was similar to the correlation between MC/20 PCR cycles/V4 primers and the theoretical mock community (Spearman correlation of 0.67, *P* = 0.071; **Figure 2**). This shows that the RBBC DNA extraction method, amplicon library generation, and MiSeq sequencing that we conducted produced bacterial relative abundance profiles that were equivalent to those produced with the procedures that were conducted by Zymo.

**FIGURE 3:**
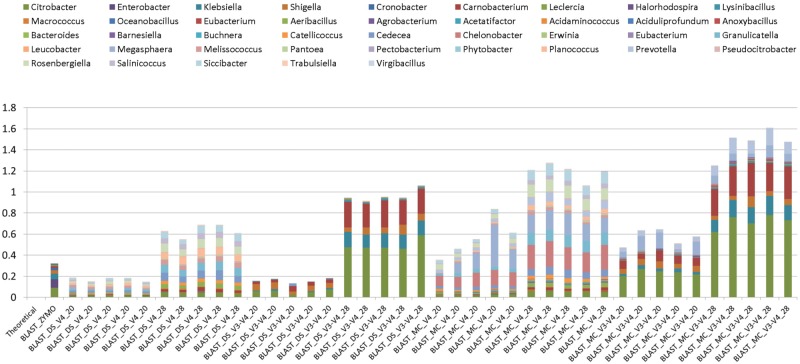
Bar chart illustrating background amplification from Zymo 16S rRNA sequencing data (Zymo), mock community cells extracted using the RBBC method (MC), and mock community DNA (DS) compared to the theoretical mock community composition. Background amplification was determined to be the presence of any genera beyond the eight expected to be present in the mock community.

Background amplification was determined to be the presence of any genera other than the eight expected to be present in the mock community. Background amplification was present in all samples including ZE (**Figure 3**). Background amplification was increased in the MC samples in comparison to the DS samples (*P* < 0.05; **Table 1** and **Figure 3**).

**Table 1:** Mean relative abundance of background amplification from mock community cells extracted using the RBBC method for DNA extraction (MC) in comparison to mock community DNA (DS) amplified with two primer sets targeting the V3–V4 and V4 region of the 16S rRNA gene at both 20 and 28 cycles.

	DNA extraction				
	DS	SEM	MC	SEM	*P*-value
BLAST_V3–V4_20	0.16	0.01	0.57	0.03	<0.05
BLAST_V3–V4_28	0.96	0.03	1.47	0.06	<0.05
BLAST_V4_20	0.17	0.01	0.56	0.08	<0.05
BLAST_V4_28	0.63	0.03	1.19	0.04	<0.05

*P-values were calculated using a Mann–Whitney U-test to evaluate background amplification from each treatment group (n = 5) resulting from DNA extraction method*.

### Primer Selection

Two primer pairs were investigated in this study; 515F/806R and Pro341F/Pro805R which targeted the V4 region and the V3–V4 region of the 16S rRNA gene, respectively. As expected, both primer sets detected eight bacterial genera (**Figure 1**) and both archaeal and bacterial species were detected in our positive control samples. The relative abundances of the genera present in the DS and the MC amplified using the two primer pairs were evaluated via correlation analysis with the theoretical mock community. The DS bacterial relative abundance profiles for V4 and V3–V4 showed strong positive correlations with the theoretical mock community profiles (*P* < 0.05; **Figure 2**). Both DS and MC amplified with V3–V4 primers showed a positive correlation to their counterpart amplified with V4 primers (*P* < 0.01; **Figure 2**).

At 28 PCR cycle, there was an increase in background amplification of both MC and DS amplified with V3–V4 primers compared to MC and DS amplified with V4 primers (*P* < 0.01; **Table 2**). Background amplification was comparable between MC and DS amplified with V3–V4 primers and MC and DS amplified with V4 primers at 20 PCR cycles (*P* > 0.5; **Table 2**).

**Table 2:** Mean relative abundance of amplification of unexpected bacteria which results from two primer sets targeting the V3-V4 and V4 region of the 16S rRNA gene which were used for amplification of mock community cells extracted with the RBBC (MC) and mock community DNA amplified at both 20 and 28 cycles.

	Primer comparison				
	V3–V4	SEM	V4	SEM	*P-*value
BLAST_DS_20	0.16	0.01	0.17	0.01	NS
BLAST_DS_28	0.96	0.03	0.63	0.03	<0.05
BLAST_MC_20	0.57	0.03	0.56	0.08	NS
BLAST_MC_28	1.47	0.06	1.19	0.04	<0.05

*P-values were calculated using a Mann–Whitney U-test to compare background amplification from each treatment group (n = 5) which results from the primer set utilized*.

### PCR Cycle Number

Two rounds of PCR were used for library preparation. The first round of PCR used primers that amplified 16S rRNA gene and the second round added the sequencing adapters and barcodes. Too many PCR cycles are thought to generate amplicon libraries that do not accurately represent microbiome composition. We tested the effect of increasing the number of PCR cycles in the first round of PCR (PCR1) from 20 PCR1 cycles to 28 PCR1 cycles. Relative abundances of taxa derived from 16S rRNA amplicon sequences from DS and MC amplified with 20 and 28 PCR cycles were compared against those of the theoretical mock community composition. The DS amplified at both 20 and 28 PCR1 cycles were significantly correlated with the theoretical mock community composition regardless of which primer pair was used (*P* < 0.05; **Figure 2**). DS and MC amplified at 20 PCR1 cycles were significantly correlated with their 28 PCR1 cycle counterpart (*P* < 0.01; **Figure 2**). There was an increase in detection of unexpected taxa at 28 PCR1 cycles in comparison to samples amplified at 20 PCR1 cycles for both DS and MC (*P* < 0.01; **Figure 3** and **Table 3**).

**Table 3:** Mean relative abundance of background amplification resulting from the number of PCR cycles during library preparation; 20 and 28 PCR cycles, constructed from DNA extracted from mock community cells extracted using the RBBC method (MC) and mock community DNA (DS) amplified with two primer sets targeting the V3–V4 and V4 region of the 16S rRNA gene.

	PCR cycle number				
	20 PCR1	SEM	28 PCR1	SEM	*P-*value
BLAST_DS_V4	0.17	0.01	0.63	0.03	<0.05
BLAST_DS_V3–V4	0.16	0.01	0.96	0.03	<0.05
BLAST_MC_V4	0.56	0.08	1.19	0.04	<0.05
BLAST_MC_V3–V4	0.57	0.03	1.47	0.06	<0.05

*P-values were calculated using a Mann–Whitney U-test to evaluate background amplification from each treatment group (n = 5) resulting from PCR cycle number*.

### Detection of Unexpected Taxa

Unexpected taxa were detected in all samples at relative abundances that varied between 0.12% and 1.6% (**Figure 3**). A higher number of PCR1 cycles (i.e., 28 PCR1 cycles versus 20 PCR1 cycles) clearly caused higher relative abundances of unexpected taxa (**Figure 3**). The highest and lowest unexpected taxa detection was in the MC_V3-V4_28 and DS_V4_20, respectively. Unexpected genera detection was one to three times higher in MC than DS in all cases when the same primer/PCR cycle number combinations were compared. This indicates that this “background noise” was influenced by the RBBC extraction method. Primers also influenced unexpected taxa detection. At 28 PCR1 cycles (but not 20 cycles), there was a significant (*P* < 0.01) increase in detection of unexpected genera in both MC and DS amplified with V3–V4 primers compared to MC and DS amplified with V4 primers. V4 primers amplified different unexpected taxa than V3–V4 (**Figure 3**).

### Sequence Classifier

Basic Local Alignment Search Tool analysis of amplicon sequences against the NCBI 16S rRNA database identified the eight bacterial genera present in the mock community from DS, MC, and ZE (**Figure 1**). The ZE, DS, and MC amplified with the V4 primers all showed significant correlation to the theoretical mock community (**Figure 2**). Classification was consistent with the theoretical composition with minimal background amplification detected (<2%; **Figure 3**). Although suitable for this experiment due to the well-defined nature and convenient number of the mock community components, BLAST in conjunction with the NCBI database is seldom used for 16S rRNA phylogenetic analysis of less well-defined communities, such as the rumen.

### OTU-Based Approach

**FIGURE 4:**
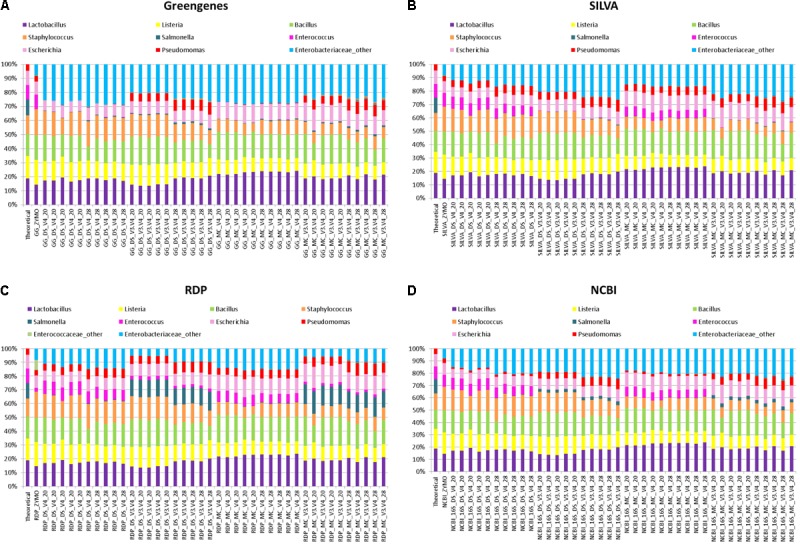
Stack plot comparing the relative abundance of the theoretical mock community compositions (Theoretical), Zymo 16S rRNA sequencing data (Zymo), mock community cells extracted using the RBBC method (MC), and mock community DNA (DS) amplified using primers targeting the V3–V4 and V4 region of the 16S rRNA gene with 20 and 28 cycles. Sequences were clustered into OTUs at 97% identify and taxonomic classification of OTUs was conducted with the RDP classifier within QIIME analyses utilizing the **(A)** Greengenes, **(B)** SILVA, **(C)** RDP, and **(D)** NCBI 16S rRNA databases.

More commonly used for taxonomic classification of 16S rRNA amplicon sequences in rumen phylogenetic studies is the generation of OTUs from sequences that share 97% identity and classification of the OTUs with the RDP classifier. This approach was investigated independently in combination with four databases: Greengenes, NCBI-16S-rRNA, RDP, and SILVA. The RDP classifier identified all eight bacterial genera from DS, MC, and ZE (**Figure 4**). However, a large proportion of the 16S rRNA OTUs that were classified as members of the Enterobacteriaceae family were not classified as far as genus level in DS, MC, and the ZE using the RDP classifier. These OTUs were assigned to “Enterobactericeae_other” (**Figure 4**). There were two genera from this family within the mock community: *Escherichia* and *Salmonella*. Representation of these two genera was inaccurate in all of the relative abundance profiles that were generated from OTUs.

*Salmonella* was identified using the RDP database for the MC (∼13%) and DS (∼12%) at 20 and 28 PCR cycles when amplified with V3–V4 primers. However, *Salmonella* was not identified for ZE (V3–V4 amplicon sequence files supplied by Zymo) using the RDP database for sequence identification despite the same region (V3–V4) of the 16S rRNA gene being amplified (**Figure 4C**). The majority of OTUs from DS and MC amplified with the V4 primers which were identified as belonging to the Enterobactericeae family with the RDP database were not classified to genus level, with less than 0.2% of sequences identified as *Salmonella* (**Figure 4C**).

Utilizing the Greengenes and NCBI databases also showed that OTUs spanning the V3–V4 region of the 16S rRNA gene increased *Salmonella* identification in comparison to the V4 sequences from MC and DS but this increase was only small and the *Salmonella* genus was severely underrepresented with both V4 and V3–V4 primers for Greengenes and NCBI (**Figures 4A,D**). *Salmonella* was equally underrepresented in all samples (<0.1%) regardless of OTU sequence length when the SILVA database was used (**Figure 4B**).

*Enterococcus* remained almost absent from the MC and DS samples identified using the Greengenes database irrespective of primer type used for amplification (**Figure 4A**). *Enterococcus* was identified in the correct proportions from the ZE (**Figure 4A**) when classified with the Greengenes database (**Figure 4A**). *Enterococcus* was also underrepresented in DS and MC samples amplified with V3–V4 primers when identified with the SILVA, RDP, and NCBI databases in comparison to DS and MC samples amplified with V4 primers and ZE (**Figures 4B–D**). *Pseudomonas* was underrepresented from DS and MC samples amplified with V4 primers when classified with the Greengenes database (<1%; **Figure 4A**).

**FIGURE 5:**
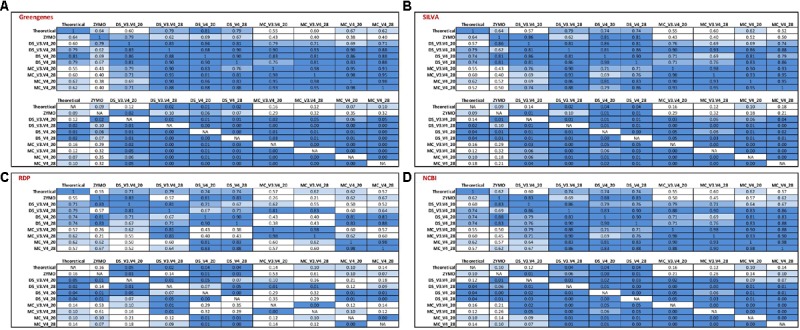
Correlation matrix of Spearman’s correlation coefficient values between the theoretical mock community compositions (Theoretical), Zymo 16S rRNA sequencing data (Zymo), mock community cells extracted using the RBBC method (MC), and mock community DNA (DS) amplified using primers targeting the V3–V4 and V4 region of the 16S rRNA gene with 20 and 28 cycles. Sequences were clustered into OTUs at 97% identify and taxonomic classification of OTUs was conducted with the RDP classifier within QIIME analyses utilizing the **(A)** Greengenes, **(B)** SILVA, **(C)** RDP, and **(D)** NCBI 16S rRNA databases. Values with a positive significant correlation are illustrated with shaded color while insignificant correlations are blank.

Spearman’s rank correlation analysis was used to compare the DS, MC, and ZE sequences classified with the RDP classifier and Greengenes, RDP, SILVA, and the NCBI 16S rRNA database (**Figure 5**). Zymo amplified their fastq files with primers targeting the V3–V4 region (which were designed by Zymo) of the 16S rRNA gene; a similar correlation between this amplified community and the theoretical mock community (*r* = 0.64, *P* < 0.1) is observed for Greengenes and SILVA databases (**Figure 5**). ZE classified with the RDP and NCBI databases gave a reduced correlation to the theoretical mock community; *r* = 0.55 and *r* = 0.62, respectively, no tendency toward the theoretical mock community was observed (**Figure 5**). The Zymo mock community gave a similar community structure regardless of database used for sequence classification (**Figure 5**). *Salmonella* was underrepresented in all of the ZE. Approximately 7% of the sequences belonging to the Enterococcaceae family were left unclassified at the genus level when classified with the RDP database (**Figure 4**).

The DS amplified with V3–V4 primers with 28 PCR1 cycles showed a similar correlation for the Greengenes, SILVA, NCBI, and RDP databases and the community compositions tended toward the theoretical mock community (*P* > 0.05; **Figure 5**). This correlation extended for the DS amplified with V3–V4 primers with 20 PCR1 cycles when sequences were classified with the RDP database (*P* < 0.05; **Figure 5C**) but not for the Greengenes, SILVA, and NCBI databases (*P* > 0.05; **Figures 5A,B,D**). All four databases showed a significant correlation for the MC and DS amplified with the V4 primers at 20 and 28 PCR1 cycles (**Figure 5**).

DNA extracted from mock community cells amplified with V4 primers with 20 PCR cycles and classified with the Greengenes database were the only condition where a tendency toward the theoretical community for sequenced MC samples was observed (*P* < 0.1). The V4 sequences amplified from DS and MC samples at 20 and 28 PCR1 cycles were significantly correlated to their V3–V4 counterparts (*P* < 0.05) when classified with using the Greengenes, SILVA, and NCBI databases (**Figures 5A,B,D**). This correlation was not apparent in the MC samples classified with the RDP database (*P* < 0.05; **Figure 5C**). For Greengenes, SILVA, and NCBI databases, DS samples were correlated with their MC counterpart amplified with identical primers with the same PCR1 cycle conditions (*P* < 0.05; **Figures 5A,B,D**). This was also observed for MC and DS samples classified with the RDP databases at 28 PCR1 cycles for both primers and for samples amplified with V4 primers at 20 PCR1 cycles (*P* < 0.05; **Figure 5C**). A significant correlation was not observed between MC and DS both amplified with V3–V4 primers at 20 PCR1 cycles when classified with the RDP database (*P* > 0.5; **Figure 5C**).

### taxMachine

**FIGURE 6:**
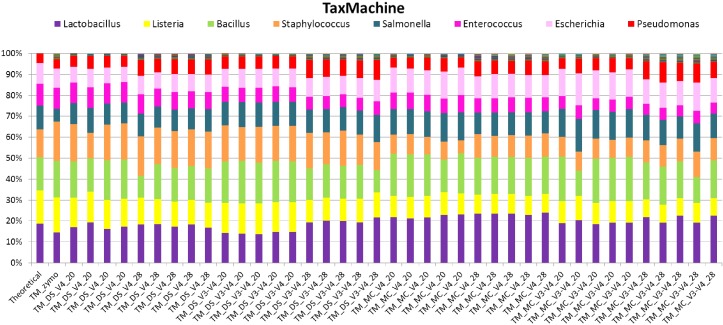
Stack plot comparing the relative abundance of the theoretical mock community compositions (Theoretical), Zymo 16S rRNA sequencing data (Zymo), mock community cells extracted using the RBBC method (MC) and mock community DNA (DS) amplified using primers targeting the V3–V4 and V4 region of the 16S rRNA gene with 20 and 28 cycles. Sequence taxonomy was classified through a high throughput taxMachine tool and contax.trim database within R.

**FIGURE 7:**
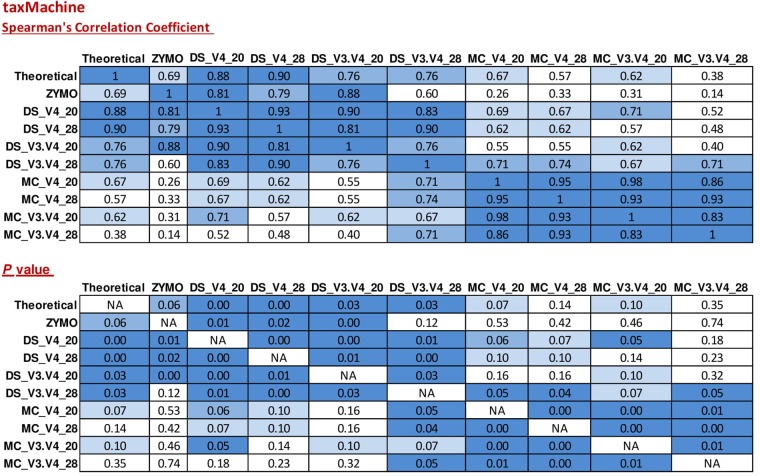
Correlation matrix of Spearman correlation coefficient values between the theoretical mock community compositions (Theoretical), Zymo 16S rRNA sequencing data (Zymo), mock community cells extracted using the RBBC method (MC), and mock community DNA (DS) amplified using primers targeting the V3–V4 and V4 region of the 16S rRNA gene with 20 and 28 cycles. Sequences were assigned taxonomic classification using the taxMachine tool and the contax.trim database within R. Values with a positive significant correlation are illustrated with shaded color while insignificant correlations are blank.

Taxonomic classification of unique sequences from DS, MC, and ZE with taxMachine identified all eight bacterial genera (**Figure 6**). Similarly to the BLAST classification, the classification was consistent with the theoretical composition. With taxMachine as taxonomic classifier, the Spearman’s correlation coefficients (**Figure 7**) were similar to that of the BLAST classification (**Figure 2**). ZE tended toward the theoretical mock community (*r* = 0.69, *P* < 0.1). The DS samples amplified with the V3–V4 and V4 at 20 and 28 PCR1 cycles were all significantly correlated with the theoretical mock community. Samples derived from DNA extracted from mock community cells showed a lower correlation to the theoretical mock community with primer choice and PCR1 cycle number influencing this correlation (**Figure 7**). MC amplified with 20 PCR1 cycles showed increased correlation to the theoretical mock community over MC amplified with 28 PCR1 cycles. DNA extracted from mock community cells and DS amplified with V4 primers showed increased correlation to the theoretical mock community over MC and DS amplified with V3–V4 primers. DNA extracted from mock community cells amplified with V4 primers with 20 PCR1 cycles tended toward the theoretical mock community.

As a comparative tool, unique sequences from ZE were also classified with a version of the RDP classifier within R package rRDP. Spearman’s correlation coefficients for unique sequences classified with RDP were significantly correlated for the theoretical mock community (*r* = 0.71, *P* < 0.05).

### Negative Controls

DNA was extracted from water as a NC and was subjected to 20 and 28 PCR1 cycles with primers targeting the V3–V4 and V4 region of the 16S rRNA gene (**Figure 3**). Compared to DS and MC, read numbers were negligible (<1000 sequences) for NC amplified with V4 primers at 20 and 28 PCR1 cycles and NC amplified with V3–V4 at 20 PCR1 cycles. One NC sample (amplified V3–V4 at 28 PCR1 cycles) had ∼14,500 merged sequences after quality filtering. Although this was much higher than the other NC samples, it was less than a 16th of the average number of sequences obtained for DS and MC community samples. NC amplified with 28 PCR1 cycles had a higher number of merged sequence reads when amplified with both V4 and V3–V4 primers.

## Discussion

Ruminants are unique in their ability to convert cellulose in plant cell walls into high-quality meat and milk protein suitable for human consumption. Despite their importance in animal protein production, ruminants are a major contributor to global anthropogenic greenhouse gas emissions by producing methane via enteric fermentation. In consideration of this, the majority of current rumen microbiomic studies aim to elucidate the microbial community present in the rumen in order to increase nutrient utilization and reduce methane emissions. It is crucial that these studies are giving an accurate representation of the rumen environment and that the studies are reproducible and suitable for meta-analysis. Currently, there is a lack of concordance between rumen microbiomic studies with research groups, including our own, failing to include negative extraction controls and positive controls in their analysis (McCabe et al., 2015; Myer et al., 2015; Meale et al., 2016; Shabat et al., 2016; Popova et al., 2017). Among the human microbiome community, concerns have been raised in response to the recent uncertainty surrounding the accuracy, consistency, and reliability of 16S rRNA phylogenetic studies (Sinha et al., 2015, 2017). In our present study, we subjected some of the methods that are currently used for investigation of the rumen microbiota using mock community cells and DSs. Power analysis inferred that technical replication of five was sufficient to deduce a significant correlation between the technical treatments examined in the current experiment and the theoretical composition of the mock community supplied by Zymo.

Zymo maintain that they provide an unbiased approach for DNA extraction. The fastq files provided by Zymo containing 16S rRNA sequencing data from their extraction of the microbial mock community with the ZymoBIOMCS^TM^ DNA Mini Kit were used initially to confirm the method of taxonomic classification. All eight genera were detected from ZE using BLAST as a method of classification, confirming that the method of classification was suitable for methodological comparative analysis. The results obtained for the ZE showed a compositional trend toward that of the theoretical mock community. 16S rRNA sequences amplified from the DS under the amplification conditions regularly utilized in rumen microbiomic studies showed better correlation to the theoretical mock community than ZE. Results from the sequenced mock community DNA indicated that both the V3–V4 and V4 primer set in this study accurately identified the genera present in the mock microbial community. All eight genera were detected and in relatively accurate proportions. The DS amplified with primers targeting the V4 region of the 16S rRNA gene showed an improved correlation to the mock microbial community than the DS samples amplified with primers targeting the V3–V4 region of the 16S rRNA gene. This may have been due to the increased length of the V3–V4 primer sequence. The base quality of the sequencing deteriorates at the end of the read (Schirmer et al., 2015). The increased length of the V3–V4 sequence allows only around 20 bp of overlap between sequence reads when a 250-cycle MiSeq reagent kit is used (Illumina had serious QC issues with the 300-cycle kits at the time of writing) and requires utilization of lower quality read ends. This may have contributed to the marginally lower correlation coefficient values discerned for the V3–V4 sequences relative to the V4 sequences. The correlation of DS to the mock community did not differ greatly between 20 and 28 PCR1 cycles. However, non-specific background amplification was increased for samples generated with 28 PCR1 cycles in comparison to amplicons generated with 20 PCR1 cycles indicating that 20 PCR1 cycles was favorable for 16S rRNA gene amplicon generation. The results provide confirmation that both primer types are suitable for amplification of bacteria for phylogenetic community analysis and that 20 PCR1 cycles was the preferable option for the amplicon amplification when utilizing these primer sets.

Having confirmed that the amplification process was satisfactory, we examined the DNA extraction methodology commonly utilized for extraction of microbial DNA from rumen microbiota. All eight genera were produced from the sequences output from the MC, inferring that the extraction protocol is suitable for both Gram-positive and Gram-negative bacteria. The MC did not reflect the theoretical mock community as precisely as the DS; however, the correlation coefficient obtained for the MC samples was similar to that obtained when comparing the ZE to the theoretical mock community. Zymo deemed this result to be an accurate representation of the theoretical mock community profile. The increase in background amplification in MC in comparison to DS may have been a contributing factor in the reduced correlation with the theoretical mock community. Addition of any steps to the full NGS protocol is invariably going to contribute to non-specific amplification, due to the sensitivity of “universal” bacterial primers (Caporaso et al., 2011; Takahashi et al., 2014) and possible residual bacterial DNA present in extraction reagents. Non-specific amplification could have been generated during the PCR step of the protocol due to the KAPA Taq DNA polymerase which is purified from *E. coli*. However, this is not likely as the number of sequence reads in the NCs was low and there was a high correlation to the theoretical mock community in the DS samples. Therefore, the reduced correlation coefficient for MC in comparison to DS is likely to be due to minor extraction inefficiencies. 16S rRNA gene amplification of DNA extraction from microbial cells would inevitably be less efficient than amplification from DNA directly; however, the DS and MC did show strong correlations to each under the same conditions highlighting the minor variation between the two amplified communities. It was also noted that MC followed the same pattern as DS, with samples amplified with V4 primers showing a higher correlation to the theoretical composition of the mock microbial community than samples amplified with primers targeting the V3–V4 region of the 16S rRNA gene, further validating the results from both the sampled MC and DS. The results provided confirmation that both primer types are suitable for amplification of bacteria for phylogenetic community analysis and that 20 PCR1 cycles was the preferable option for the amplicon amplification when utilizing these primer sets in this two PCR step protocol. The results indicate that MC provided the best simulation of the theoretical mock community when amplified with primers targeting the V4 region of the 16S rRNA gene at 20 PCR1 cycles.

The major area of concern arose not with the DNA extraction or amplicon generation process but with the taxonomic classification of the 16S rRNA sequences. NCBI BLAST in conjunction with the NCBI 16S database was chosen as a method for this assessment as the Zymo mock community being examined contained well-characterized bacteria. However, BLAST is computationally expensive, slow and not refined for the phylogenetic assessment of rumen microbiota which are largely uncharacterized to species or even family level. A more common method for handling of 16S rRNA gene sequences from rumen phylogenetic studies is the publicly available pipelines such as QIIME (Caporaso et al., 2010). QIIME is a wrapper for applications such as quality filtering, OTU picking, and taxonomic assignment. A general approach to dealing with rumen 16S rRNA gene sequences is to cluster sequences at 97% identity utilizing QIIME’s open reference-based OTU picking approach and use the RDP classifier algorithm for taxonomic classification of 16S rRNA amplicons (Wang et al., 2007). Representative OTUs are then assigned to bacterial taxonomies using RDP classifier via QIIME. This taxonomic-independent, OTU-based approach was developed to overcome the underrepresentation of 16S rRNA sequences originating in the publicly available 16S rRNA databases. This solution to cluster sequences at 97% identity was proposed by Stackebrandt and Goebel (1994) when few full-length 16S rRNA sequences were available and sequencing studies were still in their infancy. Due to the generic nature of the mock microbial community, this taxonomic-independent approach was assumed not to be a necessity but was undertaken in order to assess rumen microbiomic approaches to phylogenetic assessment. Our results indicated that the RDP classifier struggled to identify 16S rRNA V3–V4 and V4 OTUs to genus level, using the default confidence threshold of 80%. For all the databases we assessed, the RDP classifier failed to discern between the members of the Enterobacteriaceae family within ZE, DS, and MC samples when sequences were clustered into OTUs at 97% identity. It should be noted that, at a species level, the sequences of *E. coli* and *S. enterica* are two of the most well-characterized bacterial species with numerous genomic assemblies for each on the NCBI database (Edgar, 2018); reducing these sequences to representative OTU sequences may not necessarily emulate the diversity of the original sequences. It is therefore possible the confidence threshold was too high for accurate identification at a genus level.

Although not the initial aim of our study, taxonomic classification became a concern. Liland et al. (2017) highlight that while the RDP classifier tool is a good method for classification, it was not perfect and that they had found that other methods outperformed the RDP classifier (Vinje et al., 2015). A new ready-to-use tool for taxonomic classification method for 16S rRNA sequences, taxMachine, within the microclass R-package, became available during the course of our study (Liland et al., 2017). This was used to classify samples sequenced within the experiment as an alternative tool to the standard QIIME pipeline. The taxMachine tool used an internal optimized database contax.trim database for sequence classification. Liland et al. (2017) emphasized that there is no comprehensive gold standard databases available for taxonomic classification of 16S rRNA sequences due to our growing knowledge of the microbial community. This variability and lack of comprehensiveness were evident in database coverage and were visible in our own results of the RDP classification with the Greengenes, SILVA, RDP, and NCBI databases, giving varied correlation coefficient values for the comparison of ZE, DS, and MC to the theoretical mock community. The microcontax dataset was designed based on consensus taxonomy assignment from several data repositories and was highlighted to be the closest to a gold standard currently available (Liland et al., 2017). For this element of the investigation, we moved away from the convention of clustering sequences at 97% identity (Edgar, 2018). There was improvement in the community profiles from amplicon sequences derived from ZE, DS, and MC when identical sequences were aligned and classified with the taxMachine tool. Interestingly, the same result was observed when the OTU clustering step was removed and identical ZE sequences were classified with the RDP classifier within the rRDP package in R (Hahsler and Nagar, 2014). This indicates that the standard conventional pipeline we have been utilizing for 16S rRNA may not be providing the most accurate results. Clustering sequencing into OTUs of 97% identity provided a facile tool for management of the vast amount of sequence data obtained from rumen amplicon studies but it may be time to move away from this concept; however, this will need to be explored further with a mock community which more closely resembles the diversity and genera present in the rumen.

Despite the limitations in taxonomic classification highlighted in the current study, it is acknowledged that global efforts are ongoing to improve our interpretation of rumen microbial communities (Seshadri et al., 2018; Stewart et al., 2018).

## Conclusion/Future Work

Methods for phylogenetic analysis are fundamental for translation of research findings into application. This study highlights that we are following the right path for accurate community analysis; however, still have improvements to make. The analysis indicated that utilizing the RBBC method for DNA extraction in combination with primers targeting the 16S rRNA gene using 20 PCR cycles was sufficient for amplicon sequencing to generate a relatively accurate depiction of the bacterial communities present in rumen samples. However, this study was conducted using a low diversity bacterial mock community. In order to obtain a more tangible representation of the efficiency of our DNA extraction and PCR methodology, it is important to conduct a similar study using a mock community which simulates the microbiota present in the rumen. However, this is currently unavailable and common pipelines for bioinformatics analysis will also need to be appraised. Although beyond the scope of this current study, we identified some major flaws in the rumen microbiomic pipelines we and others had been utilizing. This study also highlights the need for positive mock community controls within all rumen microbiomic studies in order to discern errors which may arise at any step during an NGS protocol.

## Author Contributions

MM, EM, and SW conceived and designed the experiments. EM and MM performed the experiments and wrote the paper. EM, MM, and GB analyzed the data. EM, MM, SW, and GB contributed reagents, materials, and analysis tools, interpreted the results.

## Conflict of Interest Statement

The authors declare that the research was conducted in the absence of any commercial or financial relationships that could be construed as a potential conflict of interest.

## Footnotes

1 http://www.bioinformatics.babraham.ac.uk/projects/fastqc/

2 https://sourceforge.net/projects/bbmap/
